# Assortative mating by flowering time and its effect on correlated traits in variable environments

**DOI:** 10.1002/ece3.4765

**Published:** 2018-12-10

**Authors:** Matthew J. Rubin, Kelly M. Schmid, Jannice Friedman

**Affiliations:** ^1^ Department of Biology Syracuse University Syracuse New York

**Keywords:** assortative mating, critical photoperiod, *Erythranthe guttata*, flowering time, genetic variation, *Mimulus guttatus*, phenotypic plasticity

## Abstract

Reproductive timing is a key life‐history trait that impacts the pool of available mates, the environment experienced during flowering, and the expression of other traits through genetic covariation. Selection on phenology, and its consequences on other life‐history traits, has considerable implications in the context of ongoing climate change and shifting growing seasons. To test this, we grew field‐collected seed from the wildflower *Mimulus guttatus *in a greenhouse to assess the standing genetic variation for flowering time and covariation with other traits. We then created full‐sib families through phenological assortative mating and grew offspring in three photoperiod treatments representing seasonal variation in daylength. We find substantial quantitative genetic variation for the onset of flowering time, which covaried with vegetative traits. The assortatively‐mated offspring varied in their critical photoperiod by over two hours, so that families differed in their probability of flowering across treatments Allocation to flowering and vegetative growth changed across the daylength treatments, with consistent direction and magnitude of covariation among flowering time and other traits. Our results suggest that future studies of flowering time evolution should consider the joint evolution of correlated traits and shifting seasonal selection to understand how environmental variation influences life histories.

## 1 INTRODUCTION

The timing of plant flowering determines the biotic and abiotic environment experienced by flowers and developing seed and is critical for fitness (Elzinga et al., 2007; Inouye, 2008; Sandring & Ågren, 2009; Verhoeven, Poorter, Nevo, & Biere, 2008). Thus, flowering time is expected to be under strong selection, where phenotypic selection generally favors early flowering (Austen, Rowe, Stinchcombe, & Forrest, 2017; Munguía‐Rosas, Ollerton, Parra‐Tabla, & De‐Nova, 2011). Rapid evolution of flowering time has been demonstrated as species adapt to new environments, including changes in climatic conditions, pollinators, herbivores, or community composition (Ashworth, Walsh, Flower, Vila‐Aiub, & Powles, 2016; Ehrlén & Münzbergová, 2009; Fitter & Fitter, 2002; Franks, Sim, & Weis, 2007). In addition to demonstrating strong selection on flowering time, the ability of plant species to react quickly to selection on flowering time suggests that populations harbor substantial quantitative genetic variation for flowering responses.

Variation in flowering time within a population can cause assortative mating if individuals flowering at similar times are more likely to mate with each other than those reproducing at different times (Devaux & Lande, 2008; Weis et al., 2005). In such a situation, assortative mating by flowering time could cause temporal genetic structure or “isolation by time” (Devaux & Lande, 2008; Hendry & Day, 2005). The effect of assortative mating for the next generation depends on the heritability of flowering time and other traits that are genetically correlated with flowering time. In addition, the consequences of assortative mating depend on the consistency of the environment between years and how selection acts on fitness‐related traits through the growing season (Galloway & Burgess, 2012).

Because flowering time is part of an overall life‐history strategy, it is often correlated with other traits related to plant growth and allocation (Ehrlén, 2015). Life‐history theory predicts a trade‐off between the interval of time allocated to vegetative growth versus time allocated to flowering and maturing seed (Cohen, 1976; Kozłowski, 1992), and different environments might favor alternate strategies of the timing of growth versus reproduction (Johansson, Bolmgren, & Jonzén, 2013). In annuals, plants that delay reproduction too long in the interest of gaining resources through vegetative growth could be faced with zero fitness if the environment degrades in suitability (Franks et al., 2007; Hall & Willis, 2006). However, the consequences for perennials are less clear because investment in vegetative growth may increase their probability of surviving (and reproducing) in subsequent seasons, so that the direction and magnitude of viability and fecundity selection is important (Wadgymar, Daws, & Anderson, 2017).

Many temperate plants rely on a combination of seasonal cues to time their transition from vegetative growth to flowering. Seasonal cues that drive this transition include temperature, daylength (photoperiod), and water availability (Lempe et al., 2005; Rathcke & Lacey, 1985; Romera‐Branchat, Andrés, & Coupland, 2014). For many temperate species, the transition to flowering is dependent on a critical photoperiod, below which (in long‐day species) or above which (in short‐day species), plants do not flower (Amasino & Michaels, 2010). Although photoperiod on a given calendar date is stable across years, climate change is altering temperature and precipitation, so that photoperiod and other environmental factors become decoupled (Amano, Smithers, Sparks, & Sutherland, 2010; Anderson, 2016; Wilczek et al., 2010). Populations with fixed photoperiod cues for flowering will experience novel temperatures, while populations that respond to thermal cues will experience novel photoperiods (Wadgymar, Ogilvie, Inouye, Weis, & Anderson, 2018). In general, studies show many plant species have responded to the earlier onset of spring with earlier growth and flowering (Amano et al., 2010; Cook, Wolkovich, & Parmesan, 2012; Menzel et al., 2006) which means that plants are exposed to shorter photoperiods. What consequences will different photoperiods have on phenology, plant growth, and overall plant fitness, particularly for perennials with multiple life cycles?

In this study, we use a single perennial population of the wildflower *Mimulus guttatus* (Phrymaceae) to study the consequences of assortative mating by flowering time. Specifically, we test the consequences for plants experiencing different photoperiods, as would be the case under climate models predicting earlier onset of spring. The study addresses the following questions: (a) How much quantitative genetic variation for flowering time exists within a population? (b) How does assortative mating by flowering time affect phenology and other correlated traits in environments with different photoperiods? (c) Do individuals show plasticity to photoperiod in their phenological responses and allocation strategies? We predict that offspring from assortative mating will display alternative responses to seasonal variation, so that offspring from early flowering parents will perform best in shorter photoperiods (i.e., earlier in the season) while offspring from later flowering parents will fare better in longer photoperiods. Furthermore, because flowering is part of an overall life‐history strategy, we also predict that photoperiodic controls on flowering will affect correlated traits, and that assortative mating will exaggerate these differences. Our results indicate two main findings, first that selection on flowering time at different times of the growing season will strongly affect correlated life‐history traits, and second that shifting seasons that expose plants to different daylengths will alter the relative allocation to sexual and vegetative growth.

## 2 METHODS

### 2.1 Quantifying genetic variation in a common environment

We use the common monkey flower *Mimulus guttatus *(DC.; synonym: *Erythranthe guttata *(Fisch. ex DC.) G. L. Nesom), which is a hermaphroditic herbaceous plant widely distributed in wet sites across western North America. The species shows extensive morphological variation, with populations having either annual or perennial strategies (Lowry, Rockwood, & Willis, 2008; Pennell, 1947; Twyford & Friedman, 2015). For this study, we used a perennial population (LCC) located in northern California, near Mt. Shasta at N41.1105, W122.176. We collected open‐pollinated seed from 27 plants in early August 2013. We selected plants randomly, making sure that they were at least 100 cm apart to reduce the likelihood of sampling clones. Because *M. guttatus* retains ripe seed within their pods for many weeks, we were able to collect seed from plants that had many ripe seed pods indicative of early flowering, and from plants with only one or a few ripe pods and ongoing flowering.

To characterize the genetic variation in flowering time in the population, we grew seed from maternal families in a controlled greenhouse environment. We planted ten replicates from 27 open‐pollinated field‐collected families in 6‐cm pots filled with moist Fafard 4P growing mix and randomized pots within 13 flats. We stratified seeds in the dark at 4°C for 5 days. We then moved the pots into the greenhouse set at 21°C during the day and 18°C during the night, with a 16‐hr inductive photoperiod. We misted flats twice daily until germination and bottom watered every day for 1 hr. We randomly culled seedlings to one per pot following germination. In addition to measuring flowering time (calculated as the number of days from germination to the first open flower), we measured leaf size 4 weeks after peak germination, and the number of stolons and flowers at 4 weeks after flowering. Because seed originated from field plants, there may be some maternal effects which could inflate our estimates of genetic variance and broad‐sense heritability.

### 2.2 Assortative mating and offspring variation across seasonal photoperiods

We created 12 families (two sets of six) based on flowering time, with parents within each set chosen randomly with the provision that maternal and paternal plants were unrelated. We crossed individuals that flowered early with other early flowering individuals (E × E) and we crossed late‐flowering individuals with other late‐flowering individuals (L × L). We isolated individual plants from their neighbors to avoid inadvertent cross‐pollination. We allowed seed to ripen on the maternal plant and then collected and stored the seed at room temperature for use in the next experiment. Because the seed originated from maternal plants that experienced similar greenhouse conditions and after‐ripening environments, we assume there are minimal maternal effects in this experiment.

To investigate the effect of variable seasonal environments on the next generation's flowering time and correlated traits, we grew full‐sib families in different photoperiods. We used three growth chambers (Conviron E15) that varied in their photoperiod with daylengths of 13 hr 5 min (photoperiod on April 10th at the population's natural site), 14 hr 5 min (May 4th), and 15 hr 5 min (June 11th). The temperature in all treatments was the same at 21°C days and 18°C nights. This allowed us to isolate the effect of photoperiod under a climate change scenario where it may become decoupled from temperature.

We planted 30 replicate full‐sib plants from 6 E × E crosses and 6 L × L crosses in 6‐cm pots filled with moist Fafard 4P growing mix and stratified the seed in the dark at 4°C for 7 days. We then randomly assigned the 360 pots to the three treatments, while explicitly keeping the number of replicates per family equal in each treatment (*n* = 10). We misted pots twice daily through germination and bottom watered every day for 1 hr. Because of the split‐plot design, we attempted to minimize chamber effects by rotating the plants among the different chambers (while maintaining their assigned treatment settings) and shuffling the position of each flat within a treatment every three days. We measured plants for the same suite of traits studied in the parent generation in the greenhouse. We additionally harvested each plant for above‐ground biomass four weeks after it flowered, in two collections: primary axis (including rosette leaves and inflorescence branches and flowers) and stolons (including leaves and flowers on stolons). We dried plants for a minimum of 5 days at 95°C and weighed them. Because not all plants flowered, we collected plants that did not flower at 14 weeks, which coincided with the last harvest point of flowering plants.

### 2.3 Statistical analyses

We estimated genetic variance among maternal families (*V*_G_) and broad‐sense heritability (*H* ^2^) for each trait measured on plants grown from field‐collected seed in a common greenhouse. We used restricted maximum‐likelihood generalized linear mixed models (SAS PROC GLIMMIX for stolon number and flower number, and PROC MIXED for flowering time and leaf size; SAS Inst. 2014), with family as a random effect. To calculate genetic variance, one multiplies the family variance component by the inverse of the expected relatedness of sibling offspring (Lynch & Walsh, 1998). Because we used open‐pollinated seed, we estimated the relatedness of offspring using previous studies of mating in *M. guttatus*. Thus we assumed 40% of seed were selfed and 60% outcrossed, and that one‐third of the outcrossed seed had shared paternity (Dudash & Ritland, 1991; Ivey & Carr, 2005; Ritland & Ritland, 1989; Willis, 1993). This resulted in a calculated estimate of genetic variance as 2.5 times the family variance component (i.e., 40% selfed, 40% half‐sibs, 20% full‐sibs, *r* = 0.4). We then calculated broad‐sense heritability as the estimated genetic variance divided by the total phenotypic variance (Lynch & Walsh, 1998).

To estimate genetic correlations among traits, we used best linear unbiased prediction (BLUP) to calculate breeding values for each trait and then used Pearson's product–moment correlations. BLUPs were estimated in the previous generalized linear mixed models. We also calculated genetic correlations using a single restricted maximum‐likelihood general mixed model allowing both among‐ and within‐family variances (*V*_G_) and covariances (COV_G_) to differ between traits. The genetic correlation (*r*_G12_) between two traits denoted by 1 and 2 is COV_G12_/(*V*_G1_ *V*_G2_)^1/2^, calculated from the observational variances and covariance between traits. The two methods provide qualitatively similar estimates; however, because traits have different distributions, we present results from the former method. We also calculated phenotypic Pearson's correlations between each trait and flowering time. To examine multivariate trait strategies, we used Principal Component Analysis to investigate the associations between flowering time, leaf size, stolon number, and flower number.

For the set of plants that were used as parents for the next generation, we compared their trait values with univariate tests adjusted for multiple testing. We also used two approaches to examine multivariate trait strategies by comparing the parents’ PC1 and PC2 scores from the PCA above, and we did a multivariate analysis of variance (MANOVA in PROC GLM).

For the progeny grown in growth chambers, we examined the effect of cross (E × E vs. L × L) and photoperiod on each trait separately using a generalized linear mixed model (SAS PROC GLIMMIX for flowering proportion, flower number, and stolon number and PROC MIXED for other traits). For these models, photoperiod, cross, and photoperiod by cross interactions were considered fixed effects, while family and family by photoperiod interaction were random effects. The photoperiod by cross interaction term was nonsignificant for all traits and was subsequently removed from all models. We used restricted maximum‐likelihood (REML) to estimate the variance components of random effects. We estimated genetic variance (*V*_G_) and broad‐sense heritability (*H* ^2^) using a generalized linear mixed model, as detailed above, for each trait within each treatment. We estimated genetic variance as 2 times the family variance component (for a full‐sib design with unrelated parents and assuming no inadvertent selfing), and calculated broad‐sense heritability. We estimated family‐level BLUPs for each trait in each treatment. As in the parental population, we calculated genetic and phenotypic correlations between flowering time and all other traits in each photoperiod.

Because traits covary as part of an overall life‐history strategy, we used Principal Component Analysis to investigate the following traits: leaf size, stolon number, flower number, and biomass. We excluded flowering time from this analysis, because many plants did not flower in the shorter photoperiods. We then analyzed the first two principal components using a general linear model to examine the effect of photoperiod, cross, and their interactions on the multivariate phenotypes.

We estimated critical photoperiod as the photoperiod at which 50% of plants in a family flower, consistent with previous studies in this species (Fishman, Sweigart, Kenney, & Campbell, 2014; Friedman & Willis, 2013; Kooyers, Greenlee, Colicchio, Oh, & Blackman, 2015). Although other researchers sometimes use critical photoperiod to refer to the shortest photoperiod in which there is no delay in flowering, and ceiling photoperiod to refer to the longest photoperiod in which flowering is delayed (Giakountis et al., 2010; Pouteau et al., 2008), this terminology is unsuitable for our species where repression of flowering occurs in photoperiods below the critical photoperiod. We estimated the critical photoperiod for each family separately, with logistic regression (SAS PROC PROBIT) with a binomial error distribution. The model incorporated the number of plants that flowered (*n* = 0–10) out of the total number of replicate plants per family per treatment (*n* = 10) as the response variable and photoperiod treatment as the independent variable. For one family, only a single plant flowered and so the model could not converge on a solution. The single individual from this family that flowered was in the 14‐hr photoperiod; therefore, the slope never intercepted with 50% threshold. To investigate the relationship between critical photoperiod and life‐history strategies, we regressed family BLUPs of trait values from the 15‐hr treatment against the family's calculated critical photoperiod.

## 3 RESULTS

### 3.1 Parents in a common environment

**Figure 1:**
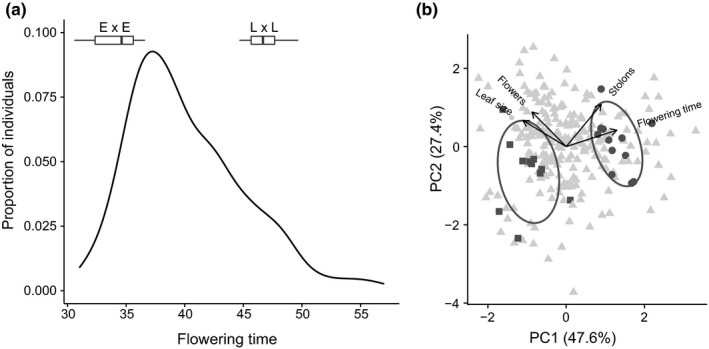
(a) Density plot of flowering time for all individuals (5 late‐flowering outliers not shown) grown in a 16‐hr photoperiod in the greenhouse. Box plots show the trait values of the selected individuals used to generate assortatively mated (E × E and L × L) offspring. (b) Principal Component Analysis showing individual positions on PC1 and PC2, and the plants that served as parents for the next generation (■ E × E, ● L × L) with ellipses representing one standard deviation around the mean of each group

Plants grown from field‐collected open‐pollinated seed in the greenhouse in a 16‐hr photoperiod showed a wide distribution of flowering time (mean = 40.7 days, *SD *= 6.1 days, range = 31.0–73.0 days, *n* = 235; Figure 1a). Maternal families differed significantly for flowering time with a broad‐sense heritability (*H* ^2^) estimate of 0.37 (Table 1). The vast majority of plants flowered (98.1%), with only five plants failing to flower over the 14‐week experiment. The nonflowering plants were from four different maternal families, and the variation was not attributed to maternal family (*Z* = 0.75, *p* = 0.23).

**Table 1:** Genetic variances (*V*_G_) and broad‐sense estimates of heritability (*H* ^2^) using a coefficient of relatedness (*r*) of 0.40 for traits measured in field‐collected seed grown under a 16‐hr photoperiod in the greenhouse

	*V*_G_	*H* ^2^	*r*_G_	*r*_P_
Flowering time	17.78 ± 7.30**	0.37	–	–
Flower number	0.17 ± 0.07**	0.34	−0.32	−0.20**
Leaf size	0.91 ± 0.31**	0.57	−0.55*	−0.46***
Stolon number	0.08 ± 0.04*	0.31	0.52**	0.54***

Note

Genetic (*r*_G_) and phenotypic (*r*_P_) correlations for each trait with flowering time.

^*^ *p < *0.05.^**^ *p < *0.01.^***^ *p < *0.001.

Maternal families also differed in leaf size, stolon number, and flower number and broad‐sense heritability estimates ranged from 0.31 to 0.57 (Table 1). There was significant genetic and phenotypic correlation between flowering time and other traits, with delayed flowering associated with smaller leaf size and larger numbers of stolons (Table 1). This is also apparent when we examine covariation in all four traits simultaneously using principal component analysis (Figure 1b). Flowering time and stolon number loaded positively on to PC1 and PC2 (PC1: 0.61 and 0.42; PC2: 0.26 and 0.67), while leaf size and flower number loaded negatively on to PC1 (−0.52 and −0.41) and positively on to PC2 (0.41 and 0.55). These results suggest that plants from different ends of the phenotypic distribution might achieve fitness through alternate routes, either reproduction through flowers or clonal growth through stolons.

We randomly selected 24 individuals as parents of assortative mating crosses from the pool of early‐ and late‐flowering plants. The E × E parents flowered an average of 13.6 days earlier than the L × L parents (Table 2, Figure 1a) and produced leaves two times larger and made fewer than half the number of stolons than the L × L parents (Table 2). The selected parents were significantly different for all measured traits except flower number, and also varied in multivariate phenotypic space (PC1 and PC2 in Table 2, and Figure 1b). Overall this suggests that assortative mating by flowering time will also affect the distribution of correlated traits, with potential implications for fitness.

**Table 2:** Means and standard errors of the E × E and L × L parents, with univariate test statistics using Bonferroni's correction for multiple tests

	Parent: E × E (mean ± *SE*)	Parent: L × L (mean ± *SE*)	*F*‐value
Flowering time	34.29 ± 0.62	47.91 ± 0.63	*F*_1,45_ = 236.41***
Flower number	32.83 ± 2.86	28.09 ± 2.54	*F*_1,45_ = 1.55
Leaf size	2.32 ± 0.17	0.78 ± 0.17	*F*_1,45_ = 42.15***
Stolon number	1.58 ± 0.26	4.25 ± 0.43	*F*_1,45_ = 26.78***
PC1	−1.40 ± 0.16	1.73 ± 0.17	*F*_1,45_ = 183.57***
PC2	−0.74 ± 0.19	0.23 ± 0.19	*F*_1,45_ = 12.81***

Notes

A MANOVA test of differences between parents for all traits simultaneously is significant (Wilks’ Lambda *F*_4,42_ = 70.00^***^).

^*^ *p < *0.05^**^ *p < *0.01^***^ *p < *0.001.

### 3.2 Offspring in variable environments

To examine the effect of assortative mating in variable environments, we grew progeny in three photoperiod treatments reflecting different onset of the growing season. The proportion of plants flowering significantly differed among photoperiod treatments and cross types (Table 3). The highest proportion of plants flowered in the 15‐hr photoperiod (91.7%), an intermediate proportion in the 14‐hr photoperiod (64.2%), and the lowest in the 13‐hr photoperiod (11.6%). Across all photoperiods, nearly three times as many progeny flowered from the E × E crosses compared to the L × L crosses (E × E: 82.7% vs. L × L: 28.3%; Table 3). A small subset of plants, around 10.2%, produced flowers only on stolons while the primary axis remained vegetative/nonreproductive. For the plants that produced exclusively stolon flowers, significantly more occurred under shorter photoperiods (*F*_182_ = 4.96, *p = *0.008) and were derived from the L × L crosses (*F*_10_ = 8.06, *p < *0.02).

**Table 3:** Summary of the influences of cross, photoperiod treatment (Trt), and maternal family on all traits measured in the offspring grown under 13‐, 14‐, and 15‐hr photoperiods in growth chambers

	Cross	Trt	Family (Cross)	Family (Cross) × Trt	13‐hr (mean ± *SE*)	14‐hr (mean ± *SE*)	15‐hr (mean ± *SE*)
Flowering proportion	4.95*	39.37***	1.92*	–	0.12 ± 0.06^a^	0.64 ± 0.14^b^	0.92 ± 0.05^c^
Flowering time	9.72*	53.93***	1.89*	–	62.47 ± 2.80^a^	50.57 ± 2.30^b^	41.09 ± 2.20^c^
Flower number	4.91	15.21***	1.54	2.50**	1.06 ± 0.53^a^	8.45 ± 4.02^b^	16.43 ± 7.81^b^
Leaf size	0.24	2.59	1.89*	2.23*	3.85 ± 0.16^a^	3.96 ± 0.16^a^	3.66 ± 0.16^a^
Stolon number	3.72	23.40***	1.32	–	6.18 ± 0.29^a^	5.09 ± 0.25^b^	4.18 ± 0.22^c^
Biomass	2.82	13.73***	1.54	2.47**	1.00 ± 0.05^a^	0.90 ± 0.05^a^	0.73 ± 0.05^b^
PC1	4.54	34.59***	1.68*	2.39**	0.92 ± 0.23^a^	0.07 ± 0.23^b^	−0.98 ± 0.23^c^
PC2	1.74	6.83**	1.94*	2.43**	0.39 ± 0.23^a^	−0.19 ± 0.23^b^	−0.20 ± 0.23^b^

Notes

Adjusted means and standard errors are reported for each photoperiod treatment; different letters indicate significant differences across photoperiods after Bonferroni's correction for multiple tests. *F*‐statistics are reported for fixed effects and Wald‐*Z *statistics for random effects. Nonsignificant terms removed from the model are indicated with “–”.

^*^p < 0.05.^**^ *p < *0.01.^***^ *p < *0.001.

In addition to the proportion of plants flowering, flowering time was significantly influenced by cross type and photoperiod (Table 3). Progeny from the E × E crosses flowered 13.1 days faster than progeny from the L × L crosses (44.8 ± 2.9 days vs. 57.9 ± 3.1 days), which is remarkably similar to the 13.6‐day difference in flowering time between the parents. Flowering time was accelerated as photoperiod increased, with plants in the 14‐ and 15‐hr photoperiods flowering earlier relative to the 13‐hr treatment by 9.6 and 17.9 days, respectively (*t*_183_ = 4.80, *p < *0.001; *t*_185_ = 8.93, *p < *0.001). Thus for plants growing early in the season under shorter daylengths, only a subset of plants will flower and/or they will take longer to transition to flowering.

**Figure 2:**
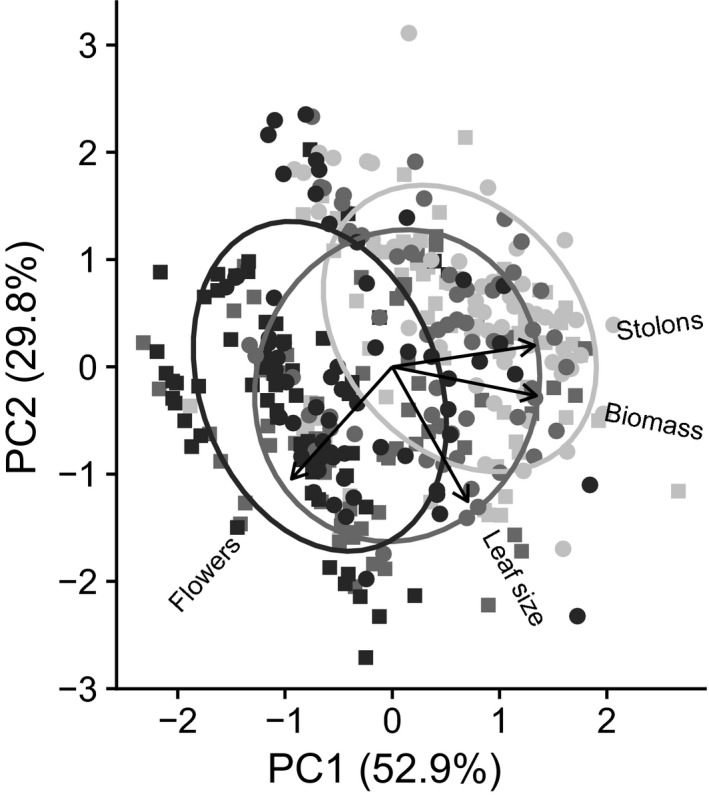
PCA plot showing PC1 and PC2 values for all offspring (■ E × E, ● L × L) grown in 13‐ (light gray), 14‐ (medium gray), and 15‐hr (dark gray) photoperiods. Ellipses represent one standard deviation around the mean of each photoperiod group

While the flowering proportion and the number of flowers increases with increasing photoperiod, the number of stolons and total biomass decreases in longer daylengths (Table 3), suggesting that later in the growing season plants allocate preferentially to sexual reproduction over clonal and vegetative growth. To examine plant life history in an integrated way, we investigated multivariate trait space using PCA. PC1 explained 52.9% of the variance and PC2 explained an additional 29.8%. Stolon number, biomass, and leaf size loaded positively onto PC1 (0.60, 0.60, 0.32, respectively), whereas flower number loaded negatively (−0.42). Leaf size, flower number, and biomass loaded positively onto PC2 (0.75, 0.63, and 0.16) and stolon number loaded negatively (−0.12). Only photoperiod explained a significant amount of variation in PC1 and PC2 (Table 3). The pattern revealed in Figure 2 is that plants in 15‐hr photoperiods have greater reproductive allocation and lower vegetative allocation, while the reverse is true for shorter photoperiods. This indicates that plastic responses to daylength can drive different life‐history strategies.

**Figure 3:**
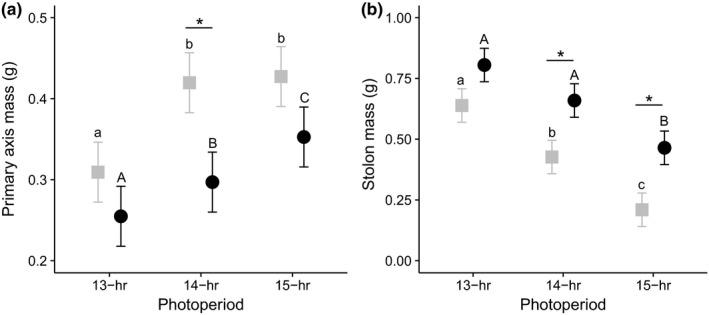
Above‐ground biomass allocation to (a) primary axis and (b) stolons for E × E (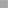) and L × L (●) offspring across 13‐, 14‐, and 15‐hr photoperiods. Significant differences between cross types within a photoperiod are denoted with an “*” (*p* < 0.05). Different letters indicate significant differences between means at *p* < 0.05, adjusted for multiple comparisons using Bonferroni's correction (lowercase letters for differences across photoperiods for the E × E crosses and uppercase letters for the L × L crosses)

To further understand how biomass allocation changes under different photoperiods, we separately examined two components of total biomass—primary axis mass and stolon mass. The E × E crosses increased allocation to the primary axis from 13 to 14 hr while continually decreasing stolon mass from 13 to 15 hr (Figure 3). The L × L crosses increased allocation to the primary axis in a stepwise fashion from 13 to 15 hr while a decrease in stolon mass was only observed from 14 to 15 hr (Figure 3). We interpret these patterns to show that allocation to clonal growth is higher in early parts of the growing season and in late‐flowering plants, and vice versa.

Consistent with the pattern observed in the parent population, flowering time was positively correlated (both genetic and phenotypic) with stolon number in all three photoperiods (Tables 1 and 4). There remained a negative phenotypic correlation between flowering time and flower number, although the genetic correlation was not significant (Table 1 and 4). However, the genetic and phenotypic correlations between flowering time and leaf size disappeared entirely, perhaps because there was very little variation in leaf size in the growth chamber experiment (see Table 3). The consistency of the correlations across the three photoperiods suggests that although correlated selection will be strong in this population, the multivariate response will be the same throughout the growing season.

**Table 4:** Genetic variances (*V*_G_) and broad‐sense estimates of heritability (*H* ^2^) using a coefficient of relatedness (*r*) of 0.5 for traits measured in the offspring generation grown under 13‐, 14‐, and 15‐hr photoperiods in growth chambers

Trt	Trait	*V*_G_	*H* ^2^	*r*_G_	*r*_P_
13‐hr	Flowering time	570.70 ± 350.78	0.89	–	–
	Flower number	1.51 ± 0.73*	0.61	−0.44	−0.77***
	Leaf size	0.36 ± 0.19*	0.48	−0.01	0.05
	Stolon number	0.04 ± 0.02*	0.51	0.59*	0.64***
	Biomass	0.07 ± 0.04*	0.60	0.43	0.32*
14‐hr	Flowering time	140.36 ± 79.56*	0.55	–	–
	Flower number	2.56 ± 1.21*	0.65	−0.35	−0.73***
	Leaf size	0.56 ± 0.26*	0.70	−0.34	0.02
	Stolon number	0.05 ± 0.02*	0.51	0.64*	0.70***
	Biomass	0.04 ± 0.02*	0.42	0.53	0.63***
15‐hr	Flowering time	137.36 ± 67.94*	0.69	–	–
	Flower number	2.13 ± 0.99*	0.70	−0.21	−0.59**
	Leaf size	0.65 ± 0.31*	0.61	0.06	0.03
	Stolon number	0.03 ± 0.02*	0.46	0.89***	0.55***
	Biomass	0.06 ± 0.03*	0.69	0.49	0.43***

Notes

Genetic (*r*_G_) and phenotypic (*r*_P_) correlations for each trait with flowering time are reported for each photoperiod.

^*^ *p < *0.05.^**^ *p < *0.01.^***^ *p < *0.001.

**Figure 4:**
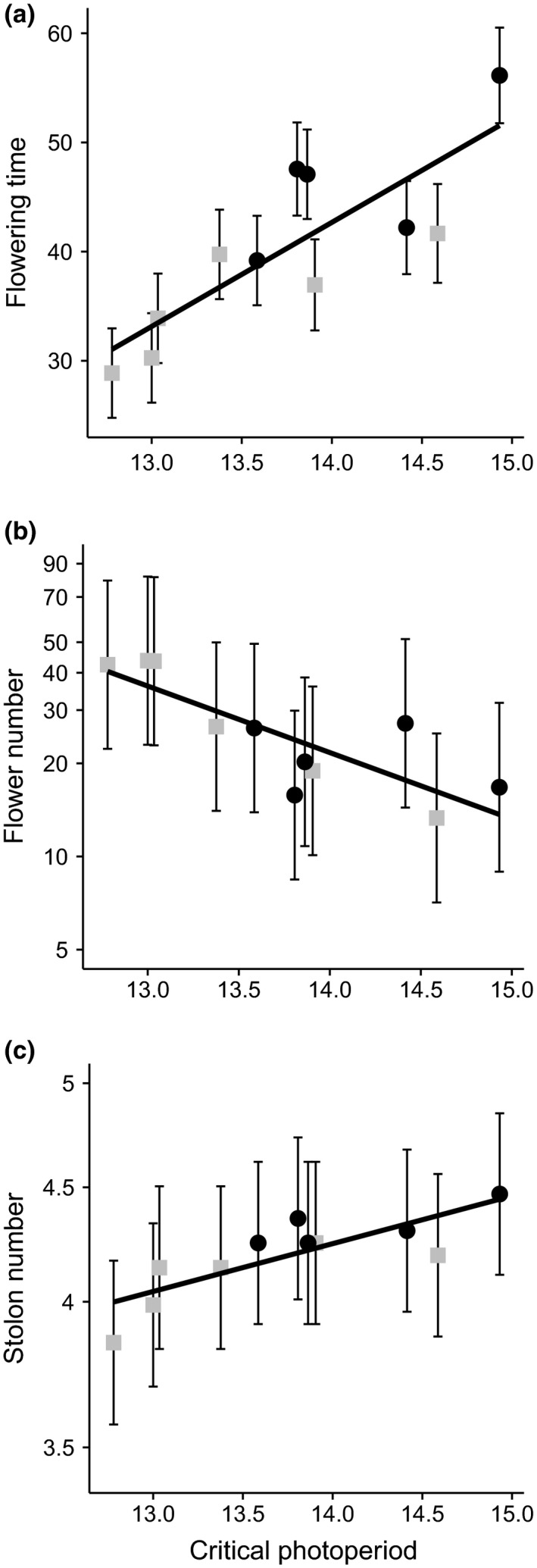
Relationship between critical photoperiod and (a) flowering time, (b) flower number, and (c) stolon number for each family (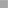 E × E, ●L × L). Means and standard errors are shown, with best‐fit linear regression lines

The critical photoperiod required for flowering varied from 12.8 to 14.9 hr among families, with one family having an even longer critical photoperiod but too few flowering plants to be estimable with our design. Critical photoperiod was associated linearly with reproductive and vegetative traits, where families with longer critical photoperiods flowered later (Figure 4a; *R* ^2^ = 0.68, *p < *0.002), produced fewer flowers (Figure 4b; *R* ^2^ = 0.82, *p < *0.03) and more stolons (Figure 4c; *R* ^2^ = 0.66, *p < *0.003). These relationships suggest that if there is selection to flower under shorter daylengths (i.e., lower critical photoperiod), this will lead to a correlated change in allocation strategies. The lack of an association between critical photoperiod and leaf size (*R* ^2^ = 0.01, *p = *0.85) or biomass (*R* ^2^ = 0.07, *p = *0.42) indicates that overall plant size does not change, but rather relative allocation changes.

## 4 DISCUSSION

We found extensive, heritable variation in flowering time within a single population of perennial *M. guttatus*. Offspring generated through assortative mating by flowering time differed in their response to photoperiod treatments. Plants flowered earlier and in higher proportion with longer daylengths and made fewer stolons and more flowers, and this pattern was consistent across assortatively mated groups. Moreover, we observed variation in critical photoperiod required for flowering of more than 2 hr within the offspring. Both plastic responses to photoperiod, and genetic differences due to parental assortative mating, determine overall life‐history strategies. The implications of this are that shifting seasons that expose plants to different daylengths will alter the relative allocation to sexual and vegetative growth, and that selection on flowering time will strongly affect correlated life‐history traits.

We have demonstrated that the onset of flowering time varied by over 40 days in seed grown from a single population in the greenhouse. Variation in flowering time in a population is expected to result in assortative mating between plants with similar flowering schedules (Breese, 1956; Devaux & Lande, 2008; Fox, 2003), and the ratio of individual variance to population variance in flowering determine the amount of assortative mating (Devaux & Lande, 2008). In a survey of 31 studies, Weis, Nardone, and Fox (2014) show that as the standard deviation of flowering date increases so does assortative mating. Given that the standard deviation in our greenhouse experiment (*SD* = 6.1) is above the median standard deviation for species included in Weis et al. (2014), it is plausible that phenological assortative mating occurs in this population in nature. There are several evolutionary consequences of phenological assortative mating, including the inflation of additive genetic variance (Breese, 1956; Felsenstein, 1981; Wright, 1921), which can facilitate more rapid response to selection (Fox, 2003). Of course this consequence requires that flowering time is heritable, our estimate of 0.37 in the parent generation is in strong agreement with an average estimate of heritability of 0.4 in a large meta‐analysis (Geber & Griffen, 2003), although this also means that over half of the assortative mating is environmental.

Even with high heritability, the expression of quantitative traits can be sensitive to different environments. For example, different QTL influence flowering time under variable field conditions or between greenhouse versus field experiments (Anderson, Lee, & Mitchell‐Olds, 2011; Dittmar, Oakley, Ågren, & Schemske, 2014; Weinig et al., 2002). Despite this, we found that the direction of trait expression in the offspring generation was consistent across the three photoperiod treatments (no significant photoperiod x cross interactions for any traits), and in the predicted direction based on parent flowering times. This consistency of trait expression across environments was similar to findings by Galloway and Burgess (2012) where plants selected for early or late flowering showed consistent responses in understory and gap environments. Furthermore, we found that the genetic correlations between flowering time and other traits were consistent across the different environments (although it was not always statistically significant). Genetic correlations between traits can constrain evolution if selection acts in opposing direction on different traits (Schluter, Price, & Rowe, 1991). The genetic correlations we find may be the long‐term response to correlational selection so that plants maintain an overall life‐history strategy of early clonal and vegetative growth followed by sexual reproduction later in the season.

The trait loadings in the principal component analysis, and the significant genetic correlation between flowering time and stolon number, imply that plants do not simultaneously invest in sexual and vegetative growth. In both parent and offspring generations, early flowering plants make few stolons, and late‐flowering plants make many stolons. Previous work showed a similar pattern across populations of annual and perennial *M. guttatus* and identified shared QTL between flowering time and stolons in a mapping population (Friedman, Twyford, Willis, & Blackman, 2015). Remarkably, within a single population, we recapitulate a large proportion of the variation in both traits. Identifying the same pattern among families within a natural population provides support that this is a genetic correlation, and that the population harbors substantial quantitative variation for these allocation traits. In our current study, we also detected significant differences across photoperiods and cross types in the proportion of biomass allocated to the primary rosette (and primary inflorescence) versus stolons (Figure 3). Plants with higher primary axis biomass are partitioning resources preferentially toward sexual reproduction; while plants with larger stolon biomass are partitioning resources toward vegetative and clonal growth. Understanding how selection maintains genotypes with different allocation patterns in this perennial population and the implications for lifetime fitness would require multiyear field experiments that compare recruitment from seed versus success of stolon rosettes. In some species, clonal reproduction leads to higher population growth than sexual reproduction (Schulze, Rufener, Erhardt, & Stoll, 2012), in other studies the two reproductive modes have equal importance (Weppler, Stoll, & Stöcklin, 2006), or the relative success of the two strategies changes temporally and spatially (Chen et al., 2015). Overall, there is increasing recognition that selection on other fitness components could be driving patterns of phenotypic selection on flowering phenology and requires careful testing (Austen et al., 2017).

Our photoperiod treatments were designed to replicate different parts of the growing season and reveal how allocation and flowering strategies vary through the season. In addition to finding strong photoperiod effects on allocation to sexual and clonal growth, we were surprised to also find wide variation in critical photoperiod (the minimum daylength required for 50% of individuals to flower) within this single population—ranging from 12.8 to over 15 hr among maternal families. Although extensive variation in critical photoperiod has been documented across populations of *M. guttatus *(Friedman & Willis, 2013) with populations at higher elevations and later growing season start dates having longer critical photoperiods (Kooyers et al., 2015), this is the first demonstration of extensive variation within a single population. The range in critical photoperiod we discovered corresponds to ~8 weeks at the population's natural location. Although we may have inflated variation for critical photoperiod through assortative mating, our results show that this population harbors extensive genetic variation for photoperiodic responses.

In a warming environment with earlier starts to the growing season, plants will experience shorter photoperiods. Our results suggest several potential outcomes to this. Selection may favor individuals with a lower critical photoperiod and more rapid flowering. Because of genetic covariation between traits, this could result in a population that shifts toward more sexual reproduction and reduced clonal growth. This would mimic the pattern of selection that likely produced the annual ecotype of *M. guttatus *(Hall & Willis, 2006). Alternatively, if the duration of the growing season increases alongside earlier snowmelt, then selection may favor plants that grow vegetatively for longer and invest more in clonal growth. Greater clonal growth may benefit population viability—in a study of 472 species, Herben, Šerá, and Klimešová (2015) found that clonality was associated with reduced mortality compared to nonclonal species, probably due to mortality risks being spread among ramets (Eriksson, 1993). Certainly the consequences of earlier snowmelt are going to be complicated. Anderson, Inouye, McKinney, Colautti, and Mitchell‐Olds (2012) show that earlier snowmelt imposes strong directional selection for early flowering in *Boechera stricta*. In a subsequent study, Wadgymar et al. (2017) refine this finding to show that while fecundity selection favors earlier flowering, viability selection favors delayed flowering and larger plant size. It is likely that similar processes occur in *M. guttatus* since there is a tradeoff between flowering early (which will provide reproductive assurance) and making stolons (which could increase survival). Certainly our results show that the effects of earlier onset of spring cannot be considered only in terms of flowering time, and our ability to understand how plants will respond to changing seasonal environments depends on disentangling the strength and direction of multivariate selection across the growing season.

## CONFLICT OF INTEREST

None declared.

## AUTHOR CONTRIBUTIONS

M.J.R, K.M.S., and J.F. participated in design and execution of the research including data collection, analysis, and interpretation, and preparation of the manuscript.

## DATA ACCESSIBILITY

Raw data have been deposited at the Dryad Digital Repository (https://doi.org/10.5061/dryad.b9j94n3).

## References

[ece34765-bib-0001] Amano, T. , Smithers, R. J. , Sparks, T. H. , & Sutherland, W. J. (2010). A 250‐year index of first flowering dates and its response to temperature changes. Proceedings of the Royal Society of London B: Biological Sciences, 277(1693), 2451–2457. 10.1098/rspb.2010.0291. PMC2894925 20375052

[ece34765-bib-0002] Amasino, R. M. , & Michaels, S. D. (2010). The timing of flowering. Plant Physiology, 154, 516–520. 10.1104/pp.110.161653 20921176 PMC2948982

[ece34765-bib-0003] Anderson, J. T. (2016). Plant fitness in a rapidly changing world. New Phytologist, 210, 81–87. 10.1111/nph.13693 26445400

[ece34765-bib-0004] Anderson, J. T. , Inouye, D. W. , McKinney, A. M. , Colautti, R. I. , & Mitchell‐Olds, T. . (2012). Phenotypic plasticity and adaptive evolution contribute to advancing flowering phenology in response to climate change. Proceedings of the Royal Society of London B: Biological Sciences, 279(1743), 3843–3852. 10.1098/rspb.2012.1051. PMC3415914 22787021

[ece34765-bib-0005] Anderson, J. T. , Lee, C. R. , & Mitchell‐Olds, T. (2011). Life‐history QTLs and natural selection on flowering time in *Boechera stricta*, a perennial relative of *Arabidopsis* . Evolution, 65, 771–787. 21083662 10.1111/j.1558-5646.2010.01175.x PMC3155413

[ece34765-bib-0006] Ashworth, M. B. , Walsh, M. J. , Flower, K. C. , Vila‐Aiub, M. M. , & Powles, S. B. (2016). Directional selection for flowering time leads to adaptive evolution in *Raphanus raphanistrum* (Wild radish). Evolutionary Applications, 9, 619–629. 27099626 10.1111/eva.12350 PMC4831463

[ece34765-bib-0007] Austen, E. J. , Rowe, L. , Stinchcombe, J. R. , & Forrest, J. R. (2017). Explaining the apparent paradox of persistent selection for early flowering. New Phytologist, 3, 929–934. 10.1111/nph.14580 28418161

[ece34765-bib-0008] Breese, E. L. (1956). The genetical consequences of assortative mating. Heredity, 10, 323–343. 10.1038/hdy.1956.30

[ece34765-bib-0009] Chen, X.‐S. , Li, Y.‐F. , Xie, Y.‐H. , Deng, Z.‐M. , Li, X. , Li, F. , & Hou, Z.‐Y. (2015). Trade‐off between allocation to reproductive ramets and rhizome buds in *Carex brevicuspis* populations along a small‐scale elevational gradient. Scientific Reports, 5, 12688. 26228352 10.1038/srep12688 PMC4521143

[ece34765-bib-0010] Cohen, D. (1976). The optimal timing of reproduction. American Naturalist, 110, 801–807. 10.1086/283103

[ece34765-bib-0011] Cook, B. I. , Wolkovich, E. M. , & Parmesan, C. (2012). Divergent responses to spring and winter warming drive community level flowering trends. Proceedings of the National Academy of Sciences, 23, 9000–9005. 10.1073/pnas.1118364109 PMC3384199 22615406

[ece34765-bib-0012] Devaux, C. , & Lande, R. (2008). Incipient allochronic speciation due to non‐selective assortative mating by flowering time, mutation and genetic drift. Proceedings of the Royal Society of London B: Biological Sciences, 275, 2723–2732. 10.1098/rspb.2008.0882 PMC2605824 18700202

[ece34765-bib-0013] Dittmar, E. L. , Oakley, C. G. , Ågren, J. , & Schemske, D. W. (2014). Flowering time QTL in natural populations of *Arabidopsis thaliana* and implications for their adaptive value. Molecular Ecology, 23, 4291–4303. 25039363 10.1111/mec.12857

[ece34765-bib-0014] Dudash, M. R. , & Ritland, K. (1991). Multiple paternity and self‐fertilization in relation to floral age in *Mimulus guttatus* (Scrophulariaceae). American Journal of Botany, 78, 1746–1753. 10.1002/j.1537-2197.1991.tb14539.x

[ece34765-bib-0015] Ehrlén, J. (2015). Selection on flowering time in a life‐cycle context. Oikos, 124, 92–101. 10.1111/oik.01473

[ece34765-bib-0016] Ehrlén, J. , & Münzbergová, Z. (2009). Timing of flowering: Opposed selection on different fitness components and trait covariation. American Naturalist, 173, 819–830. 10.1086/598492 19335224

[ece34765-bib-0017] Elzinga, J. A. , Atlan, A. , Biere, A. , Gigord, L. , Weis, A. E. , & Bernasconi, G. (2007). Time after time: Flowering phenology and biotic interactions. Trends in Ecology & Evolution, 22, 432–439. 10.1016/j.tree.2007.05.006 17573151

[ece34765-bib-0018] Eriksson, O. (1993). Dynamics of genets in clonal plants. Trends in Ecology and Evolution 8, 313–316. 21236180 10.1016/0169-5347(93)90237-J

[ece34765-bib-0019] Felsenstein, J. (1981). Continuous‐genotype models and assortative mating. Theoretical Population Biology, 19, 341–357.

[ece34765-bib-0020] Fishman, L. , Sweigart, A. L. , Kenney, A. M. , & Campbell, S. (2014). Major quantitative trait loci control divergence in critical photoperiod for flowering between selfing and outcrossing species of monkeyflower (*Mimulus*). New Phytologist, 201, 1498–1507. 24304557 10.1111/nph.12618

[ece34765-bib-0021] Fitter, A. , & Fitter, R. (2002). Rapid changes in flowering time in British plants. Science, 296, 1689–1691. 10.1126/science.1071617 12040195

[ece34765-bib-0022] Fox, G. A. (2003). Assortative mating and plant phenology: Evolutionary and practical consequences. Evolutionary Ecology Research, 5, 1–18.

[ece34765-bib-0023] Franks, S. J. , Sim, S. , & Weis, A. E. (2007). Rapid evolution of flowering time by an annual plant in response to a climate fluctuation. Proceedings of the National Academy of Sciences, 104, 1278–1282. 10.1073/pnas.0608379104 PMC1783115 17220273

[ece34765-bib-0024] Friedman, J. , Twyford, A. D. , Willis, J. H. , & Blackman, B. K. (2015). The extent and genetic basis of phenotypic divergence in life history traits in *Mimulus guttatus* . Molecular Ecology, 24, 111–122. 25403267 10.1111/mec.13004 PMC4657477

[ece34765-bib-0025] Friedman, J. , & Willis, J. H. (2013). Major QTLs for critical photoperiod and vernalization underlie extensive variation in flowering in the *Mimulus guttatus* species complex. New Phytologist, 199, 571–583. 23600522 10.1111/nph.12260

[ece34765-bib-0026] Galloway, L. F. , & Burgess, K. S. (2012). Artificial selection on flowering time: Influence on reproductive phenology across natural light environments. Journal of Ecology, 100, 852–861. 10.1111/j.1365-2745.2012.01967.x

[ece34765-bib-0027] Geber, M. A. , & Griffen, L. R. (2003). Inheritance and natural selection on functional traits. International Journal of Plant Sciences, 164, S21–S42. 10.1086/368233

[ece34765-bib-0028] Giakountis, A. , Cremer, F. , Sim, S. , Reymond, M. , Schmitt, J. , & Coupland, G. (2010). Distinct patterns of genetic variation alter flowering responses of *Arabidopsis* accessions to different daylengths. Plant Physiology, 152, 177–191. 10.1104/pp.109.140772 19889880 PMC2799355

[ece34765-bib-0029] Hall, M. C. , & Willis, J. H. (2006). Divergent selection on flowering time contributes to local adaptation in *Mimulus guttatus* populations. Evolution, 60, 2466–2477. 10.1554/05-688.1 17263109

[ece34765-bib-0030] Hendry, A. P. , & Day, T. (2005). Population structure attributable to reproductive time: Isolation by time and adaptation by time. Molecular Ecology, 14, 901–916. 10.1111/j.1365-294X.2005.02480.x 15773924

[ece34765-bib-0031] Herben, T. , Šerá, B. , & Klimešová, J. (2015). Clonal growth and sexual reproduction: Tradeoffs and environmental constraints. Oikos, 124, 469–476. 10.1111/oik.01692

[ece34765-bib-0032] Inouye, D. W. (2008). Effects of climate change on phenology, frost damage, and floral abundance of montane wildflowers. Ecology, 89, 353–362. 18409425 10.1890/06-2128.1

[ece34765-bib-0033] Ivey, C. T. , & Carr, D. E. (2005). Effects of herbivory and inbreeding on the pollinators and mating system of *Mimulus guttatus *(Phrymaceae). American Journal of Botany, 92, 1641–1649. 10.3732/ajb.92.10.1641 21646081

[ece34765-bib-0034] Johansson, J. , Bolmgren, K. , & Jonzén, N. (2013). Climate change and the optimal flowering time of annual plants in seasonal environments. Global Change Biology, 19, 197–207. 10.1111/gcb.12006 23504731

[ece34765-bib-0035] Kooyers, N. J. , Greenlee, A. B. , Colicchio, J. M. , Oh, M. , & Blackman, B. K. (2015). Replicate altitudinal clines reveal that evolutionary flexibility underlies adaptation to drought stress in annual *Mimulus guttatus* . New Phytologist, 206, 152–165. 25407964 10.1111/nph.13153

[ece34765-bib-0036] Kozłowski, J. (1992). Optimal allocation of resources to growth and reproduction: Implications for age and size at maturity. Trends in Ecology & Evolution, 7, 15–19. 10.1016/0169-5347(92)90192-E 21235937

[ece34765-bib-0037] Lempe, J. , Balasubramanian, S. , Sureshkumar, S. , Singh, A. , Schmid, M. , & Weigel, D. (2005). Diversity of flowering responses in wild *Arabidopsis thaliana* strains. PLoS Genetics, 1, e6 10.1371/journal.pgen.0010006 PMC1183525 16103920

[ece34765-bib-0038] Lowry, D. B. , Rockwood, R. C. , & Willis, J. H. (2008). Ecological reproductive isolation of coast and inland races of *Mimulus guttatus* . Evolution, 62, 2196–2214. 18637837 10.1111/j.1558-5646.2008.00457.x PMC11110535

[ece34765-bib-0039] Lynch, M. , & Walsh, B. (1998). Genetics and analysis of quantitative traits. Sunderland, MA: Sinauer.

[ece34765-bib-0040] Menzel, A. , Sparks, T. H. , Estrella, N. , Koch, E. , Aasa, A. , Ahas, R. , … Chmielewski, F. M. (2006). European phenological response to climate change matches the warming pattern. Global Change Biology, 12, 1969–1976. 10.1111/j.1365-2486.2006.01193.x

[ece34765-bib-0041] Munguía‐Rosas, M. A. , Ollerton, J. , Parra‐Tabla, V. , & De‐Nova, J. A. (2011). Meta‐analysis of phenotypic selection on flowering phenology suggests that early flowering plants are favoured. Ecology Letters, 14, 511–521. 10.1111/j.1461-0248.2011.01601.x 21332621

[ece34765-bib-0042] Pennell, F. W. (1947). Some Hitherto undescribed Scrophulariaceae of the Pacific states. Proceedings of the Academy of Natural Sciences of Philadelphia, 99, 155–199.

[ece34765-bib-0043] Pouteau, S. , Carré, I. , Gaudin, V. , Ferret, V. , Lefebvre, D. , & Wilson, M. (2008). Diversification of photoperiodic response patterns in a collection of early‐flowering mutants of *Arabidopsis* . Plant Physiology, 148, 1465–1473. 10.1104/pp.108.127639 18799658 PMC2577249

[ece34765-bib-0044] Rathcke, B. , & Lacey, E. P. (1985). Phenological patterns of terrestrial plants. Annual Review of Ecology and Systematics, 16, 179–214. 10.1146/annurev.es.16.110185.001143

[ece34765-bib-0045] Ritland, C. , & Ritland, K. (1989). Variation of sex allocation among eight taxa of the *Mimulus guttatus* species complex (Scrophulariaceae). American Journal of Botany, 12, 1731–1739. 10.1002/j.1537-2197.1989.tb15163.x

[ece34765-bib-0046] Romera‐Branchat, M. , Andrés, F. , & Coupland, G. (2014). Flowering responses to seasonal cues: What's new? Current Opinion in Plant Biology, 21, 120–127. 10.1016/j.pbi.2014.07.006 25072635

[ece34765-bib-0047] Sandring, S. , & Ågren, J. (2009). Pollinator‐mediated selection on floral display and flowering time in the perennial herb *Arabidopsis lyrata* . Evolution, 63, 1292–1300. 19154392 10.1111/j.1558-5646.2009.00624.x

[ece34765-bib-0048] Schluter, D. , Price, T. D. , & Rowe, L. (1991). Conflicting selection pressures and life history trade‐offs. Proceedings of the Royal Society of London B: Biological Sciences 246, 11–17.

[ece34765-bib-0049] Schulze, J. , Rufener, R. , Erhardt, A. , & Stoll, P. (2012). The relative importance of sexual and clonal reproduction for population growth in the perennial herb *Fragaria vesca* . Population Ecology, 54, 369–380. 10.1007/s10144-012-0321-x

[ece34765-bib-0050] Twyford, A. D. , & Friedman, J. (2015). Adaptive divergence in the monkey flower *Mimulus guttatus* is maintained by a chromosomal inversion. Evolution, 69, 1476–1486. 25879251 10.1111/evo.12663 PMC5029580

[ece34765-bib-0051] Verhoeven, K. , Poorter, H. , Nevo, E. , & Biere, A. (2008). Habitat‐specific natural selection at a flowering‐time QTL is a main driver of local adaptation in two wild barley populations. Molecular Ecology, 17, 3416–3424. 10.1111/j.1365-294X.2008.03847.x 18573164

[ece34765-bib-0052] Wadgymar, S. M. , Daws, S. C. , & Anderson, J. T. (2017). Integrating viability and fecundity selection to illuminate the adaptive nature of genetic clines. Evolution Letters, 1, 26–39. 10.1002/evl3.3 30283636 PMC6121800

[ece34765-bib-0053] Wadgymar, S. M. , Ogilvie, J. E. , Inouye, D. W. , Weis, A. E. , & Anderson, J. T. (2018). Phenological responses to multiple environmental drivers under climate change: Insights from a long‐term observational study and a manipulative field experiment. New Phytologist, 218, 517–529. 10.1111/nph.15029 29451307

[ece34765-bib-0054] Weinig, C. , Ungerer, M. C. , Dorn, L. A. , Kane, N. C. , Toyonaga, Y. , Halldorsdottir, S. S. , … Schmitt, J. (2002). Novel loci control variation in reproductive timing in *Arabidopsis thaliana* in natural environments. Genetics, 162, 1875–1884. 12524356 10.1093/genetics/162.4.1875 PMC1462365

[ece34765-bib-0055] Weis, A. , Nardone, E. , & Fox, G. (2014). The strength of assortative mating for flowering date and its basis in individual variation in flowering schedule. Journal of Evolutionary Biology, 27, 2138–2151. 10.1111/jeb.12465 25186618

[ece34765-bib-0056] Weis, A. , Winterer, J. , Vacher, C. , Kossler, T. , Young, C. , & LeBuhn, G. (2005). Phenological assortative mating in flowering plants: The nature and consequences of its frequency dependence. Evolutionary Ecology Research, 7, 161–181.

[ece34765-bib-0057] Weppler, T. , Stoll, P. , & Stöcklin, J. (2006). The relative importance of sexual and clonal reproduction for population growth in the long‐lived alpine plant *Geum reptans* . Journal of Ecology, 94, 869–879.

[ece34765-bib-0058] Wilczek, A. , Burghardt, L. , Cobb, A. , Cooper, M. , Welch, S. , & Schmitt, J. (2010). Genetic and physiological bases for phenological responses to current and predicted climates. Philosophical Transactions of the Royal Society of London B: Biological Sciences, 365, 3129–3147. 10.1098/rstb.2010.0128 20819808 PMC2981944

[ece34765-bib-0059] Willis, J. H. (1993). Partial self‐fertilization and inbreeding depression in two populations of *Mimulus guttatus* . Heredity, 71, 145–154. 10.1038/hdy.1993.118

[ece34765-bib-0060] Wright, S. (1921). Assortative mating based on somatic resemblance. Genetics, 6, 144–161. 17245960 10.1093/genetics/6.2.144 PMC1200503

